# Genetic screening analysis of patients with hereditary diffuse gastric cancer from northern and northeastern Brazil

**DOI:** 10.1186/1897-4287-12-18

**Published:** 2014-08-13

**Authors:** Caroline Aquino Moreira-Nunes, Mariceli Baia Leão Barros, Bárbara do Nascimento Borges, Raquel Carvalho Montenegro, Leticia Martins Lamarão, Helem Ferreira Ribeiro, Amanda Braga Bona, Paulo Pimentel Assumpção, Juan Antonio Rey, Giovanny Rebouças Pinto, Rommel Rodriguez Burbano

**Affiliations:** 1 Biological Science Institute, Federal University of Para, Belem, PA 66075110, Brazil; 2 Center of Hematology and Hemotherapy of Para – HEMOPA Foundation, Belem, PA 66033000, Brazil; 3 Biotecnology Institute, Federal Rural University of Amazon, Belem, PA 66077830, Brazil; 4 Nucleus of Research in Oncology, Federal University of Para, Belem, PA 66073000, Brazil; 5 Molecular Neuro-oncogenetics Laboratory, Research Unit-Unidad de Investigación, Hospital Universitario La Paz, 28046 Madrid, Spain; 6 Genetics and Molecular Biology Laboratory, Federal University of Piaui, Parnaiba, PI 64049-550, Brazil

**Keywords:** Array comparative genomic hybridization, aCGH, Hereditary diffuse gastric cancer, CDH1, HDGC

## Abstract

**Background:**

Hereditary diffuse gastric cancer (HDGC) is a hereditary autosomal inherited syndrome associated with *CDH1* germline mutations. In Brazil, gastrointestinal tumors are among the most prevalent tumor types and constitute a serious public health problem, especially in the northern and northeastern regions. This study aimed to investigate germline mutations, methylation pattern and genomic rearrangements in the *CDH1* gene and quantitative changes in the DNA of HDGC patients in northern and northeastern Brazil.

**Methods:**

Twenty-seven DNA samples from the members of four families affected by HDGC were analyzed using array comparative genomic hybridization (aCGH), DNA sequencing and methylation pattern.

**Results:**

No evidence of gain and loss events or any rearrangements were found in any of the samples tested using aCGH. No promoter region hypermethylation was observed either. Two of the four families presented different types of germline mutations. The 185G > T and 1018A > G germline mutations detected in this study have been described in Asian and European families, respectively. The ancestors of the two families carrying these mutations had originated from those continents.

**Conclusion:**

This is the first study to evaluate *CDH1* gene germline mutations in Brazilian families with HDGC. In our study, 50% of the families showed no *CDH1* gene alterations, and it is possible that in regions with a high incidence of gastric cancer, such as northern and northeastern Brazil, environmental factors might have induced the different genetic alterations analyzed in this study.

## Introduction

In northern and northeastern Brazil, gastric cancer (GC) is the second most common type of cancer and is considered a serious public health problem because it is usually diagnosed at an advanced stage [1]. In addition to its sporadic manifestation, GC is also associated with various syndromes that predispose the carrier to cancer. Among the familial forms of GC, hereditary diffuse gastric cancer (HDGC) is the only form with a well-defined genetic cause [2,3].

In order for a family to qualify for a diagnosis of HDGC, the following criteria must be met: two or more documented cases of diffuse gastric cancer in first- or second-degree relatives with at least one diagnosed before the age of 50 years or three or more cases of documented diffuse gastric cancer in first- or second-degree relatives independent of the age of onset [4,5].

The correlation between an E-cadherin gene germline mutation (*CDH1* inactivation) and the predisposition to diffuse gastric cancer was first identified in a large family in New Zealand [6]. Based on this finding, HDGC was characterized and other occurrences were described in patients with different ethnic backgrounds [7,8].

The *CDH1* gene, located on the 16q22.1 chromosome, encodes the E-cadherin intercellular adhesion protein; this protein acts as a tumor suppressor and plays an important role in maintenance of the epithelial tissue architecture [9]. Mutations in this gene primarily affect both the intracellular and extracellular domains of the protein and thus affect the integrity of the protein, leading to disturbances in epithelial tissue cell-cell adhesion, increased cell motility and an enhanced infiltrative capacity and tumor metastasis [10,11]. As HDGC is an infiltrative tumor, endoscopy and biopsy-based diagnostic strategies are inefficient; furthermore, given the high penetrance of *CDH1* mutations, prophylactic gastrectomy is recommended for affected patients [11,12].

Over one hundred germline mutations have been described for the *CDH1* gene. Although several of these mutations have been detected in different families, to date, no hotspot has been characterized [3,13]. Although the *CDH1* gene is the predominantly affected gene in HDGC, other inactivation mechanisms should be investigated. Additionally, large deletions might also be responsible for *CDH1* inactivation [8,14-20]. For this reason, an array comparative genomic hybridization (aCGH) analysis is essential to the identification of quantitative alterations in the *CDH1* gene or other genome regions in families affected by HDGC [21].

The objective of this study was to identify germline mutations in the *CDH1* gene and/or quantitative genomic alterations in four families with HDGC in northern and northeastern Brazil.

## Methodology

### Patients

The samples evaluated in this study were obtained from patients who fulfilled the clinical criteria according to the latest consensus of the International Gastric Cancer Linkage Consortium [5]. The patients had been treated at referral hospitals in northern and northeastern Brazil. The samples were processed in their states of origin and then sent to the Federal University of Pará (Universidade Federal do Pará; UFPA) for genetic analysis.

A retrospective study of the family members was also conducted to identify previous generations with DGC. All genetically analyzed patients (or their guardians) signed consent forms that had been approved by the Research Ethics Committee of University Hospital João de Barros Barreto (Hospital Universitário João de Barros Barreto; HUJBB) under protocol number 274/12. Assurance was provided that the use of biological materials and participation in the study would not cause any harm to nor have any negative influence on patient treatment.

All biological materials except one sample (that was Formaldehyde Fixed-Paraffin Embedded tissue) was blood DNA. And all genetic screening analysis - mutation, deletion and methylation - were all looking for germline changes, not somatic.

### DNA extraction

Peripheral blood samples were collected in EDTA-containing tubes and extracted with the QIAamp® DNA Blood Mini Kit (Qiagen N.V., Venlo, The Netherlands) according to the manufacturer’s instructions.

Paraffin block sample was obtained from one patient, and extracted with the QIAamp DNA® FFPE Tissue Kit (Qiagen) according to the manufacturer’s instructions.

### Genotypic analysis

The obtained DNA was used to perform molecular *screening* of the *CDH1* gene. Gene fragments containing the analyzed polymorphisms were amplified by polymerase chain reaction (PCR) using gene-specific reaction conditions and primers (Table 1) according to Brooks-Wilson *et al*. [22].

**Table 1:** Primer sequences used in the study and their annealing temperatures

**Exon**	**Forward 5′- 3′**	**Reverse 5′- 3′**	**Tm (°C)**
1	M13F GTGAACCCTCAGCCAATCAG	M13R TGACGACGGGAGAGGAAG	63
2	M13F TGTTGGTTTCGGTGAGCAG	M13R GGTGT3GGGAGTGCAATTTCT	61
3	M13F CGCTCTTTGGAGAAGGAATG	M13R AACGGTACCAAGGCTGAGAA	58
4	M13F GCTGTCTGGCTAGGTTGGAC	M13R TTTTCCCTTTCTCTCCTTGG	58
5	M13F GAAAGGGAAAAGACCCAGTG	M13R GGATCCAGCATGGGTTGAC	58
6	M13F GCCCCTTCTCCCATGTTT	M13R CTTTGGGCTTGGACAACACT	56
7	M13F GGGCAGAATTGGATTAAGCA	M13R TGTCCACGGGATTGAGCTA	57
8	M13F CTGGGTAGGCCAAAGGT	M13R CCATGAGCAGTGGTGACACTT	57
9	M13F AATCCTTTAGCCCCCTGAGA	M13R AGGGGACAAGGGTATGAACA	61
10	M13F CCAAAAGCAACAGTTAAGGA	M13R CAAATGACAAAATGCCATGA	56
11	M13F AGCGCTTAAGCCGTTTTCA	M13R GAGGGGCAAGGAACTGAACT	60
12	M13F AAGGCAATGGGGATTCATTA	M13R ATTGAAAGGTGGGGATCTGG	59
13	M13F CAATTTTATTCTGGAATGAGCTTTT	M13R CAGGAAATAAACCTCCTCCATTT	55
14	M13F GCTGCTTCTGGCCTTCTTA	M13R GCTGTTTCAAATGCCTACCTCT	55
15	M13F TGAACATAGCCCTGTGTGTATG	M13R TTTTGACACAACTCCTCCTG	58
16	M13F AGACTTCTTGCCCCAGATGA	M13R AACCACCAGCAACGTGATTT	63

Forward primers have – 21M13F tail attached for sequencing: TGTAAAACGACGGCCAGT; reverse primers have M13R tail attached for sequencing: CAGGAAACAGCTATGAC.

The result of each reaction was subjected to direct sequencing on an ABI 3130 capillary sequencing platform (Applied Biosystems/Life Technologies, Carlsbad, CA, USA). The sequences were obtained as electropherograms and analyzed with the software package provided with the equipment. The generated sequences were analyzed with the BIQ Analyzer software package [23].

### aCGH

High-density probe microarray analyses were performed to determine the copy number variation (CNV). The complete genomes of all patients were evaluated with the goal of identifying genes related to tumor development.

The *Affymetrix® CytoScan™HD Array* (Affymetrix, Inc., Santa Clara, CA, USA) was used; this system features a total of approximately 1.9 million probes for detecting CNV and 750,000 SNP molecular markers. The standard protocol incorporates the following eight procedures before scanning the chip: genomic DNA digestion, NSP adapter ligation, fragment amplification by PCR (Polymerase Chain Reaction), PCR product purification, PCR product fragmentation, end-labeling, hybridization and washing.

The *Chromosome Analysis Suite* software v1.2.1 (*Affymetrix®*) was used for the chip analysis.

### Methylation status

For methylation analyses, the samples were subjected to DNA modification using sodium bisulfite [24]. A fragment with 22 CpGs of the *CDH1* promoter region was amplified using a nested PCR strategy [25]. Fragments obtained were purified using PCR Purification Kit (Invitrogen/Life Technologies, Carlsbad, CA, USA) and sequenced using an ABI3130 automatic sequencer (Applied Biosystems, Foster City, CA, USA). The sequences were aligned with BioEdit v7.0.5 [26]. Methylation analyses were run in BiQ Analyzer [23] software.

### Immunohistochemistry

An anti-cadherin (Abcam PLC, Cambridge, UK) commercial primary antibody was used to detect the protein product of the *CDH1* gene (HDGC). Streptavidin-biotin-peroxidase staining as described by Hsu *et al*. [27] was adopted as the immunohistochemical method. The normality parameter was defined with samples from normal (non-tumor) formalin-fixed and paraffin-embedded tissues that had been obtained from routine samples. The World Health Organization histopathological classifications for each tumor were used as well as the Lauren classification for gastric cancer [28].

## Results

The detailed patient data are shown in Table 2. A total of 27 patient samples were collected for genetic analysis from four unrelated families with histories of DGC in northern and northeastern Brazil. All biological samples collected were peripheral blood, except for the patient AM05, whose sample was Paraffin-embedded tumor. The sample comprised 18 men and nine women with a mean age of 42 years and an age range of 20–75 years (24–56 years for women and 20–75 years for men).

**Table 2:** Identification of the patients analyzed in this study

**Patient**	**Gender**	**Age at diagnosis**	**Type of cancer**
**(Family A)**			
**^AM05**	male	36	*DGC
**AM06**	female	56	none
**AM07**	male	31	*DGC
**AM08**	female	26	none
**(Family B)**			
**P05**	male	29	*DGC
**P06**	male	27	*DGC
**P07**	male	23	none
**P08**	male	20	none
**(Family C)**			
**C05**	male	75	Prostate
**C14**	male	52	*DGC
**C15**	male	50	*DGC
**C16**	female	49	*DGC
**C17**	female	48	none
**C18**	female	45	none
**C19**	female	47	none
**C20**	male	25	none
**C21**	male	23	none
**C22**	female	42	none
**C23**	female	24	none
**C24**	male	56	none
**C25**	male	35	none
**C26**	male	31	none
**C27**	male	58	none
**C28**	female	31	none
**(Family D)**			
**M08**	female	67	*DGC
**M09**	male	62	*DGC
**M10**	male	64	none

**^-** Paraffin-embedded tumor sample.

* - Diffuse Gastric Cancer.

**Figure 1:**
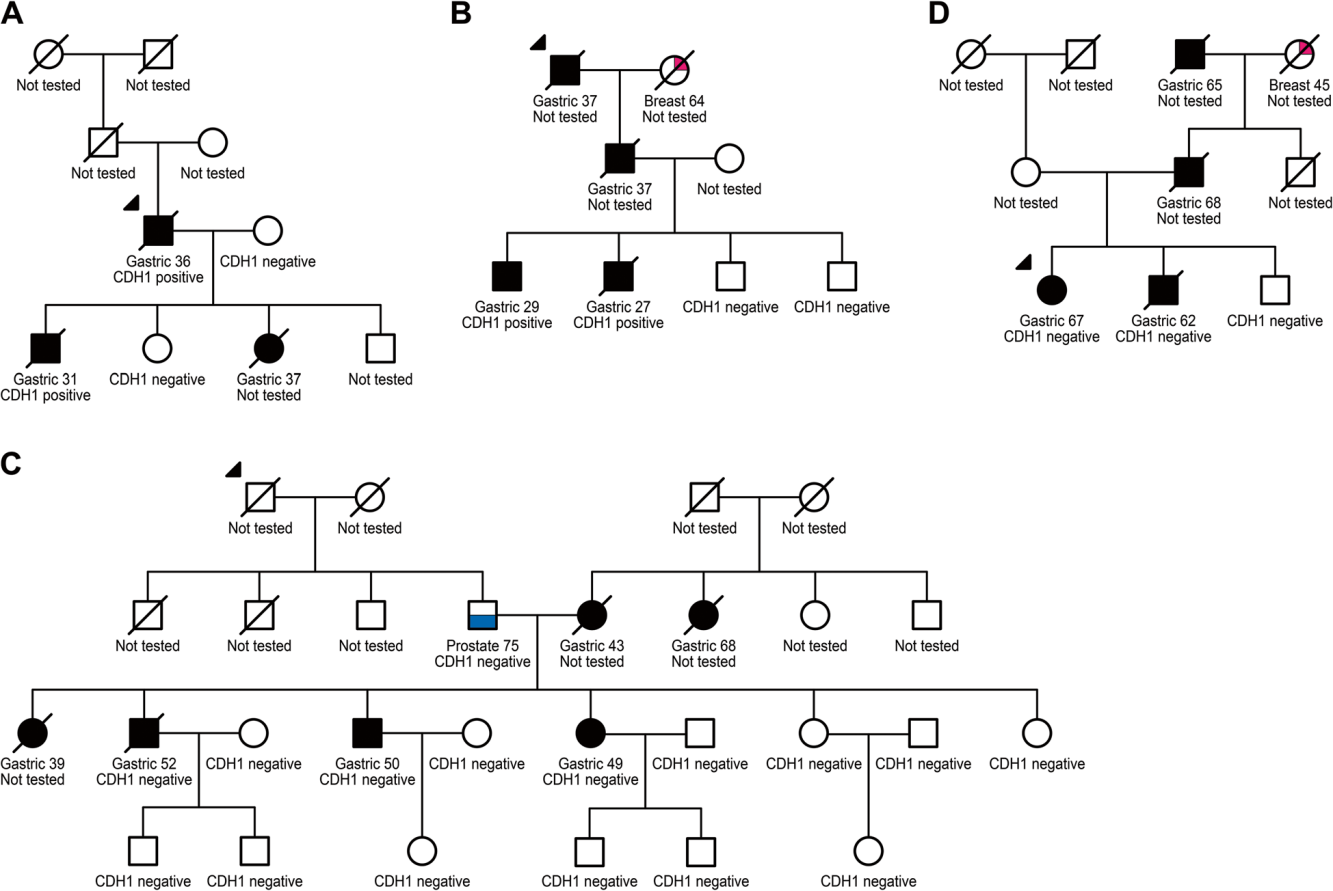
**Pedigrees of hereditary diffuse gastric cancer families. (A)**, **(B)**, **(C)** and **(D)** represents the four families presented in this study. The numbers present under the symbols represent the age at diagnosis. The solid symbols represent the affected members with confirmed diffuse gastric cancer diagnoses. Upper left arrows indicate the probands.

Of these 27 patients, nine (33.4%) had been previously diagnosed with diffuse gastric cancer, whereas the remaining patients did not have any type of gastric tumor. Among the patients diagnosed with DGC, the age at diagnosis ranged from 27–67 years.After the retrospective study and using information collected from the analyzed patients, it was possible to identify relatives with a history of DGC and other types of tumors (Figure 1).

### Mutations in the *CDH1* gene

Among the four families of patients, two families exhibited germline mutations in the *CDH1* gene, namely family A from northern Brazil and family B from northeastern Brazil (Table 3). In the other two families, which originated from northeastern Brazil, no mutations were found in the evaluated *CDH1* exons.

**Table 3:** **Germline *CDH1 *** **mutations identified in hereditary diffuse gastric cancer families**

**Family ID**	**Patient ID**	**Age at onset/sex**	**Exon**	**Base changes**	**Amino acid change**	**Mutation consequence**
A	AM05	36/male	3	185 G > T	Gly ⋄ Val	Missense
	AM07	31/male				
B	P05	29/male	8	1018 A > G	Thr ⋄ Ala	Missense
	P06	27/male				

### Array comparative genomic hybridization (aCGH)

The aCGH sample analysis showed no gains or losses of DNA in the four tested families (27 samples).

### Immunoreactivity in HDGC

CDH1 protein detection was performed only for patient AM05, a carrier of the germline mutation 185 G > T and member of family A. This patient died before the start of the study, and a genetic analysis was performed on a paraffin-embedded tumor tissue. The other study participants with this syndrome donated peripheral blood for genetic analysis, and consequently, there was no need to request paraffin blocks of the resected tumor tissues. The tumor cells of patient AM05, revealed negative immunoreactivity to the E-cadherin protein.

### Methylation status in HDGC

We analyzed the methylation pattern of a *CDH1* gene promoter region fragment in one CpG island containing 22 CpG dinucleotides, to find possible heritable epimutations. No promoter region hypermethylation was observed in all 27 tested individuals, from the four families, and also in 10 healthy controls.

## Discussion

In addition to sporadic manifestation, gastric cancer can be associated with various syndromes that predispose the carrier to cancer. Among the familial forms of gastric cancer, HDGC is the only syndrome with a well-defined genetic cause [2,3].

The relationship between a genetic germline mutation in the E-cadherin-encoding gene (*CDH1* inactivation) and the predisposition to HDGC was first identified in a family in New Zealand [6]. Based on this finding, the HDGC syndrome was characterized, and other occurrences were described in patients with different ethnic backgrounds [29].

Mutations in *CDH1* gene affect protein integrity, thus causing disturbances in cell-cell adhesion in epithelial tissues, increasing cell motility and enhancing infiltrative behavior of the tumor and metastasis development [10,11].

This genetic study of four families that carried the HDGC syndrome allowed us to evaluate a previously unaddressed issue within the Brazilian population. *CDH1* germline mutations are observed in 30–40% of cases that meet the clinical criteria for HDGC [30,31]. In our study, 50% of the families exhibited *CDH1* gene mutations. This information is useful in clinical evaluations of patients with family histories of HDGC because in addition to prophylactic measures, the information facilitates monitoring aimed at identifying the occurrence of other tumors such as lobular carcinomas of the breast; these types of cancers had also been detected in members of the families analyzed in this study [13,20,32].

The cases described in the literature demonstrate that the most common *CDH1* gene alterations associated with HDGC are point mutations and small changes in the reading frame; these occur in approximately 93% of families with *CDH1* gene mutations [13,20,33].

In this study, two individuals in family A, a father and son (Figure 1A), were carriers of a familial *CDH1* gene mutation at nucleotide position 185 (185G > T); this mutation causes a change in the protein structure from the amino acid glycine to valine. This *missense* mutation was first described by Shinmura *et al.*[34] in a family of Japanese origin. Our results suggest the hypothesis that this 185G > T mutation was introduced into family A by the grandfather of the proband (first generation pedigree), who was of Japanese origin, and suggest that this mutation might result from this ethnic background.

A *CDH1* gene germline mutation was also identified in two brothers from family B who had developed an early form of gastric cancer (before 45 years of age) [35]. The mutation was identified at nucleotide position 1018 (1018A > G) and resulted in a change from the amino acid threonine to alanine. The 1018A > G mutation was previously described in a family of European origin and in a Chinese family [36,37]. Because the proband of this family was of Portuguese descent, it is possible that their ancestors brought this mutation to Brazil upon immigration. On the other hand, another alternative to ancestral mutations is an increased susceptibility of different *CDH1* locations to mutations.

A literature review revealed the occurrence of a total of 122 germline mutations in the *CDH1* gene in HDGC [38]. Although several types of mutations have been detected in different families, no hotspot has been characterized to date [3,13]; this led us to sequence all 16 exons of the gene in the 30 members of the four analyzed families.

Of the mutations reported in the literature, approximately 15% are shared by many families worldwide, suggesting that *CDH1* mutation-associated HDGC might share a common ancestry, as was suggested for families A and B in this study [33,39].

In the other two families (C and D), in which the evaluated subjects carried no *CDH1* gene mutations, there were considerable numbers of HDGC-affected members in different generations (Figure 1). Pinheiro et al. [20] have proposed that many HDGC families carry some mutations in non-exonic regulatory regions of *CDH1* (or in upstream regulators). Based on this, it may be possible in the future to test families C and D, for allele-specific loss on *CDH1*.

In other populations in which the incidence of gastric cancer is high, there are also reports of HDGC-carrier families with large numbers of affected individuals who do not carry *CDH1* gene germline mutations. This finding might be consequent to exposure to environmental risk factors or the susceptibility of individuals to genetic alterations in low-penetrance genes [40,41].

In a study conducted in southeastern Brazil on 88 patients with early gastric cancer, 16 of whom had familial histories of gastric cancer, it was possible to detect changes in the E-cadherin protein expression in 41 individuals via immunohistochemical analysis. Although *CDH1* gene sequencing was not conducted to identify possible mutations, the immunohistochemical analysis revealed the involvement of *CDH1* in the early development of gastric cancer in a Brazilian population [42].

Because the *CDH1* gene acts as a tumor suppressor, other gene inactivation mechanisms of this gene besides germline mutations in one allele should be investigated to identify changes to the second allele in somatic cells (second event of the Knudson hypothesis [43]), which was not able to be done in the present work. Mechanisms such as *CDH1* gene deletion [33] and promoter region hypermethylation [44], were investigated in germline from those analyzed patients, because are also considered potentially responsible for its inactivation.

Cytosine hypermethylation in the CpG dinucleotides present within the *CDH1* promoter region induces transcriptional silencing [45] and might thus explain the lack of E-cadherin immunoreactivity. However, analyses to confirm the presence of *CDH1* gene promoter region hypermethylation in patients with HDGC have not been conclusive. For example, studies by Li *et al*. [46] and Concolino *et al.*[47] demonstrated *CDH1* promoter hypermethylation-mediated gene silencing in 53–57% of patients with HDGC. Conversely, Wu *et al*. [48] reported an absence of this phenomenon in 140 Chinese gastric cancer patients with a familial history of HDGC.

The analyses conducted in the present study did not detect *CDH1* gene promoter region hypermethylation in the families with HDGC syndrome. Our results reinforce the theory put forth by Yamada *et al.*[19] who, after analyzing 22 Japanese patients with early gastric cancer, suggested that germline *CDH1* promoter hypermethylation was not a predisposing factor of gastric cancer.

In addition to epigenetic alterations in the promoter region, the *CDH1* gene might also suffer deletions even in the absence of point mutations in the germlines of patients with HDGC [49]. Kim *et al*. [50] used multiplex ligation-dependent probe amplification (MLPA) to analyze 23 patients with HDGC and, similarly to our study, found no deletions or duplications in these genes.

Oliveira *et al.*[33] genetically analyzed 160 patients with HDGC from different geographic regions and found that *CDH1* gene deletions occurred in the peripheral blood in approximately 4% of families affected by this syndrome. The same authors also observed that all families with *CDH1* deletions originated from countries with low gastric cancer incidence rates. A large number of patients with HDGC, in northern and northeastern from Brazil, needs to be analyzed to verify if the absence of *CDH1* gene deletions and other quantitative genomic alterations, as found in patients of this work, corroborate this observation because these regions have high gastric cancer incidence rates.

It is widely known that the most common forms of cancer develop as a result of interactions between endogenous and environmental factors such as the diet [51]. Environmental factors might be related to the high incidence of these neoplasms in the northern and northeastern regions, or for some reason the HDGC frequency is higher than elsewhere in the world, where sporadic gastric cancer is also endemic, such as Japan and Korea. In particular, *Helicobacter pylori* (*H. pylori*) infection during childhood as well as the high consumption of salt-preserved foods, infrequent use of refrigeration and low consumption rates of cereals, fresh fruits and vegetables are considered risk factors for gastrointestinal tumors [52-55].

## Conclusions

This is the first study to evaluate *CDH1* gene germline mutations and quantitative gene alterations in Brazilian families with HDGC. *CDH1* gene germline mutations were found in 50% of the families evaluated. The detected mutations appeared to originate from Asia and Europe. This migratory flow does not rule out the possibility that environmental factors might have caused these same mutations in the affected members of the analyzed families. No quantitative changes were observed in the genomes of any of the analyzed families. It is possible that in regions with high gastric cancer incidence rates such as northern and northeastern Brazil, environmental factors or other molecular mechanisms might induce different genetic alterations from those analyzed in this study.

## Competing interests

The authors declare that they have no competing interests.

## Authors’ contributions

Conceived and designed the experiments: CFAMN, MBLB, BNB, LML, RMRB. Performed the experiments: CFAMN, MBLB, BNB, LML, ABB. Analyzed the data: CFAMN MBLB, BNB, HFR, RMRB. Wrote the paper: CFAMN, PPA, JAR, GRP, RMRB. All authors read and approved the final manuscript.
